# Enhanced Trapping of Yellowjacket Wasps (Hymenoptera: Vespidae) via Spatial Partitioning of Attractants

**DOI:** 10.3390/insects8010017

**Published:** 2017-02-06

**Authors:** Dangsheng Liang, Jose E. Pietri

**Affiliations:** Apex Bait Technologies, Inc., Santa Clara, CA 95054, USA; jose@apexbait.com

**Keywords:** heptyl butyrate, yellowjacket, wasp, attractant, synergy, spatial, trap

## Abstract

Several yellowjacket species are important pests in both their native habitat and in areas where they are invasive. Traps that contain one or more chemical attractants to lure insects inside are commonly used to combat these yellowjackets in urban environments. Usually, attractants are placed within the trap and combined indiscriminately, though little is known about how this design influences trap attractiveness or efficacy. Here, using the common attractant heptyl butyrate in combination with chicken extract, we demonstrate that spatial partitioning of attractants results in increased capture of the western yellowjacket *Vespula pensylvanica*—a widespread pestiferous species. Specifically, we show that partitioning of these attractants results in increased visitation of yellowjackets to a trap while also leading to more individuals entering the trap. Further, we provide evidence that this effect is driven by the ability of heptyl butyrate to function as an attractant to the general location of the trap while also blocking the effects of meat extract as a trap-entering stimulus. Thus, our data challenge the current paradigm of combining attractants inside yellowjacket traps, and suggest that these methods can be improved through the consideration of spatial variables and interactions. Our results not only provide novel insight into the mechanisms of yellowjacket attraction, but are also likely to be applicable to the control of other insects for which attractant-based traps are used.

## 1. Introduction

Several yellowjacket species, such as the western yellowjacket (*Vespula pensylvanica*), are widespread pests in urban environments, often building large nests near man-made structures [1]. This proximity to human dwellings makes them not only a nuisance, but a threat to public health. In mid-to-late summer, western yellowjacket nests can undergo exponential growth [2], and the accidental disturbance of a nest by an unsuspecting individual can result in multiple stings from guards defending the nest. In addition, foragers searching for food outside the nest often come into contact with people at outdoor events, increasing the potential for stings. In a given year, stings from hymenoptera—including yellowjackets—lead to thousands of hospital visits [3]. Though yellowjacket stings are not usually life-threatening, they are known to be quite painful, and can cause severe reactions in a small proportion of the human population [4]. Stings may also pose a significant threat to pets and native wildlife in areas where yellowjackets are an invasive species [5,6].

Controlling populations of pestiferous yellowjackets such as *V. pensylvanica* is difficult and laborious due to the size and location of nests. However, the use of traps against these yellowjackets is a common method of reducing their interactions with humans. Usually, these traps contain one or more chemical attractants. Frequently, this includes synthetic heptyl butyrate (HB), which was the first yellowjacket attractant to be commercialized and is highly attractive to *V. pensylvanica* [7]. While traps containing HB are effective at reducing the number of foraging workers in a particular location [8], they have not been shown to reduce sting incidence or populations over a larger area [8,9]. A better understanding of HB attraction could lead to improved trapping that may alleviate these issues.

The natural sources of HB and the mechanism underlying its attractiveness to yellowjackets remain largely unknown. Possibly, HB is an analog of natural fruity esters such as hexyl butyrate, which is emitted by ripening apples [10]. This volatile and other structurally similar chemicals are highly attractive to several yellowjacket species [11], and may act as olfactory cues used by foraging workers to find rotting fruit—a key source of carbohydrates in the field. Interestingly, acetic acid—another olfactory cue produced by fermenting fruit—can enhance the attractiveness of HB to some yellowjackets, demonstrating the potential for synergistic attraction between odors from carbohydrate sources [12]. In addition to rotting fruit, several yellowjacket species—including *V. pensylvanica*—also forage for carrion (meat) and small insect prey using visual and olfactory cues [13]. Meat-based products are frequently used in yellowjacket baits and traps against these species [14,15,16,17]. While very little is known about how HB interacts with attractants from meats, recent studies suggest that HB may synergize with protein-derived attractants. For instance, compounds from honey, fermenting brown sugar, scale insect honeydew, and mussels outcompete known attractants when combined [18,19]. Similarly, combinations of acetic acid and volatiles associated with insect prey (hexenal or linalool) synergistically interact [20].

Exploiting interactions between HB and other attractants may be one way to improve the efficacy of traps containing HB against *V. pensylvanica* and other yellowjacket species with similar behavior. Indeed, most commercially available yellowjacket traps recommend the addition of meat or other food products to act as additional attractants inside traps containing HB. However, this suggestion is made despite a lack of understanding regarding the mechanisms by which HB acts alone or in concert with protein-derived attractants.

Given this knowledge gap, we sought to examine the spatial interactions between the commonly used yellowjacket attractant heptyl butyrate and protein-derived attractants. Surprisingly, our results revealed that while HB is an attractant to the general location of a trap, it does not efficiently promote trap entering. In addition, HB also interacts negatively with meat attractants, leading to reduced trap efficacy when the two are combined. However, we found that spatial partitioning of HB and chicken extract abrogates the negative interaction between them and results in more efficient trapping than when attractants are used alone or combined without spatial separation.

## 2. Materials and Methods

### 2.1. Yellowjacket Traps

The traps used in these studies were Rescue brand yellowjacket traps purchased from supermarkets (Sterling International Inc., Spokane, WA, USA). The predominant species of yellowjacket in the region where experiments were conducted is the western yellowjacket, *Vespula pensylvanica*. Our trap catches consisted almost exclusively of this species, and the numbers reported include only this species.

### 2.2. Attractants

Heptyl butyrate (HB) was purchased from Sigma-Aldrich (St. Louis, MO, USA). Unless otherwise specified, 50 µL was loaded onto cotton balls and either placed inside the base of the trap or hung outside of the trap, depending on the experimental setup. Chicken meat extract was used as a short distance attractant, as chicken meat has been shown to be among the preferred protein sources for foraging *V. pensylvanica* [17]. Extract was prepared by submerging small pieces of cooked chicken meat in dichloromethane for 10 min and then rinsing twice with additional solvent. The extract was partitioned with water to concentrate volatile organic material and remove excess water-soluble components. The dichloromethane fraction was then concentrated under a stream of nitrogen gas. This chicken extract was applied to filter paper at a concentration of 1 g of chicken meat equivalent and placed within the base of the trap.

### 2.3. Yellowjacket Field Studies

For trapping tests in the field, traps were hung approximately six feet high either together on different branches of the same tree or apart on different trees >20 feet in distance. Traps were observed for 3–6 h at intervals of 5, 10, and 30 min after placement and before termination of the study. At each time point, the number of yellowjackets (*V. pensylvanica*) inside the trap as well as those hovering around the trap were counted and recorded. For each condition, a replicate consisted of a single trap at a particular location. Tests were independently replicated in paired fashion four to nine times at parks located in the City of Santa Clara, CA, USA between late morning and early afternoon during the month of October. For each experiment, the specific time points and replicates are stated in the figure legend.

### 2.4. Data Analysis

Our experiments examined the ability of HB and chicken extract to lure yellowjackets when alone or combined inside traps, as well as when spatially separated inside and outside the trap. In brief, we analyzed the number of yellowjackets hovering around traps as well as those caught inside traps when attractants were placed inside either alone or together. Further, we measured the same variables when HB was placed outside traps containing chicken extract. The number of yellowjackets trapped or hovering for each condition was pooled for statistical comparison of the mean and variance using GraphPad Prism Version 5 (Graphpad Software, La Jolla, CA, USA). For experiments consisting of only two conditions, means were compared using a two-tailed paired *t*-test. If experiments consisted of three or more conditions, results were analyzed using repeated measures ANOVA followed by Newman–Keul’s post-test to determine differences between each group. For each test, differences were considered statistically significant if the *p*-value was less than 0.05. For each test, the particulars are described in the results section.

## 3. Results

### 3.1. Comparison of Heptyl Butyrate and Chicken Extract as Attractants

**Figure 1:**
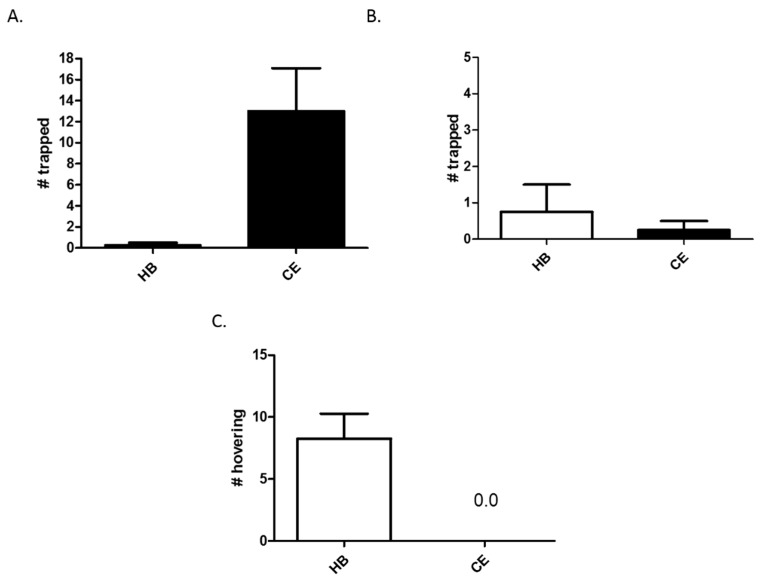
Comparison of heptyl butyrate and chicken extract as attractants in traps. Yellowjacket traps containing either heptyl butyrate (HB) or chicken extract (CE) were hung either together for 6 h (**A**) or at separate locations for 3 h (**B**,**C**). The number of yellowjackets trapped (**A**,**B**) or hovering near the trap were then counted (**C**). Each experiment consisted of a trap pair, and experiments were independently replicated four times. Data from replicates were pooled for analysis by two-tailed paired *t*-test. Bars represent the mean ± SEM. Differences were considered statistically significant (**A**,**C**) when *p* < 0.05.

In order to examine the interactions between HB and chicken extract, we first tested their attraction capacity individually. To do so, traps containing either HB (50 μL) or chicken extract (1.0 g) were hung together (Figure 1A) or separately (Figure 1B). When hung together, traps containing chicken extract performed significantly better than those containing HB, catching an average of 13 yellowjackets in 6 h, as compared to only 0.25 (*n* = 4, *p* = 0.04) in traps baited with HB. However, when the two traps were hung at separate locations, the difference between them disappeared and both performed poorly, with traps containing HB trapping an average of 0.75 yellowjackets in 3 h, and traps containing chicken extract catching an average of 0.25 within the same time (*n* = 4). Interestingly, despite not effectively luring yellowjackets inside the traps, HB was still able to attract yellowjackets to the general area of the trap more effectively than chicken extract (*n* = 4, *p* = 0.02). That is, 3 h after the placement of traps in separate locations, an average of eight yellowjackets were observed hovering near traps containing HB, while none were observed near traps containing chicken extract (Figure 1C).

### 3.2. Effects of Heptyl Butyrate on the Attractiveness of Chicken Extract

**Figure 2:**
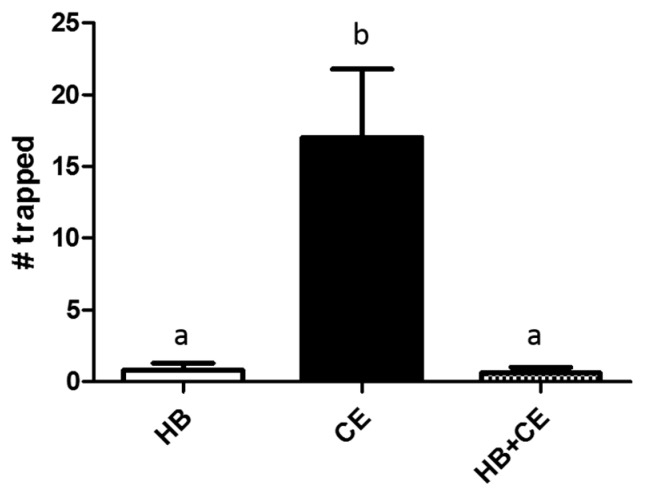
Effects of heptyl butyrate on the attractiveness of chicken extract in traps. Yellowjacket traps containing either heptyl butyrate (HB), chicken extract (CE), or a combination of the two (HB + CE) were hung together for 3 h. The number of yellowjackets trapped by each was then counted. The experiment was independently replicated at five different locations, and bars represent the mean ± SEM. Data from replicates were pooled for analysis by one-way repeated measures ANOVA followed by Newman-Keuls post-test. Differences were deemed statistically significant when *p* < 0.05, and are indicated by letters in the graph (same letters = no difference).

We next sought to examine whether HB and chicken extract could increase yellowjacket trapping when combined. To test this possibility, we hung traps containing either HB alone (50 μL), chicken extract alone (1.0 g), or a combination of HB and chicken extract (Figure 2). When these traps were hung together for 3 h, traps containing chicken extract outperformed those containing HB alone, as expected from our previous results (Figure 1). However, we were surprised to find that traps containing both HB and chicken extract performed equally as poorly as those containing HB alone, and were outperformed by those containing only chicken extract (*n* = 5, *p* = 0.0001). While traps containing chicken extract caught an average of 17 yellowjackets in 3 h, those containing HB alone caught only 0.8, and those containing HB and chicken extract caught 0.6.

### 3.3. Partitioning of Heptyl Butyrate and Chicken Extract Results in Increased Trapping

**Figure 3:**
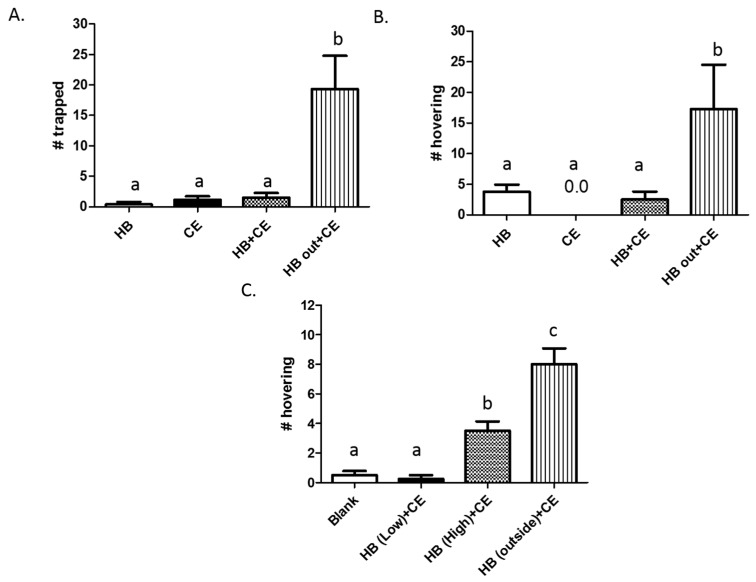
Partitioning of heptyl butyrate and chicken extract results in increased trapping. Traps containing heptyl butyrate (HB), chicken extract (CE), or a combination of both inside (HB + CE) were compared against traps containing CE inside and HB outside. Traps were hung at separate locations for 3 h, and the number of yellowjackets caught (**A**) or hovering around the trap (**B**) were counted. In (**C**), traps containing CE inside with low (5 μL) or high (50 μL) doses of HB inside, or HB on the outside (50 μL) were hung at separate locations for 3 h, and the number of yellowjackets hovering in the vicinity were counted. Experiments were independently replicated at four to six different locations, and bars represent the mean ± SEM. Data from replicates were pooled for analysis by one-way repeated measures ANOVA followed by Newman–Keuls post-test. Differences were deemed statistically significant when *p* < 0.05, and are indicated by letters in the graph (same letters = no difference).

We hypothesized that if HB negatively interacts with meat extracts when combined, its use in traps could be enhanced by placing it outside of the trap while including meat extract inside the trap. We tested this hypothesis by separately hanging traps containing HB only (50 μL), chicken extract only (1.0 g), HB and chicken extract, or chicken extract on the inside with HB outside (Figure 3). Once again, traps containing HB or chicken extract only performed poorly, catching an average of 0.4 and 1.6 yellowjackets, respectively, in 3 h. Similarly, traps containing both HB and chicken extract caught an average of 1.5 yellowjackets within the same time. However, when chicken extract and HB were partitioned, traps caught an average of 19.3 yellowjackets in the same period of time. This increase was statistically significant (*n* = 6, *p* = 0.003). Similar results were obtained when the number of yellowjackets hovering around each trap were analyzed (Figure 3B). No yellowjackets were observed hovering around traps containing chicken extract only, while those containing HB had an average of 3.75 nearby. Meanwhile, traps containing both ingredients attracted an average of 2.5 yellowjackets, and traps in which attractants were partitioned attracted a significantly higher number of 17.25 (*n* = 4, *p* = 0.028).

In related experiments, we tested the effects of HB dose on the attractiveness of traps by using low (5 μL) or high (50 μL) of HB inside or outside traps in combination with chicken extract (1.0 g) on the inside (Figure 3C). In these studies, blank traps and traps containing a low dose of HB with chicken extract attracted the lowest number of yellowjackets, with an average of 0.5 and 0.25 seen hovering 3 h after hanging. Traps containing a high dose of HB with chicken extract inside the trap attracted a significantly higher number (3.5), while traps containing chicken extract inside and a high dose of HB outside attracted the highest number of eight (*n* = 4, *p* = 0.0001).

## 4. Discussion

In the present work, we demonstrate that partitioning of HB and meat-derived attractants results in increased capture of yellowjackets. To our knowledge, this is the first analysis of spatial interactions between yellowjacket attractants. Further, we provide a mechanism for this phenomenon by revealing the ability of HB to block trap-entering stimuli. These findings are completely novel, and significantly advance our understanding of yellowjacket attraction while encouraging the consideration of spatial variables involved in the use of chemical attractants for insect trapping.

Chemical attractants of insects can be broadly divided into two categories: those that act over long-range and those that act over short-range, though these distances are vaguely defined in the literature [21]. Usually, long-range attractants are volatile chemicals that act through the air to signal insects toward a particular location, while short-range attractants typically trigger a particular behavior (e.g., landing, feeding, etc.). Our data demonstrate a similar distinction between HB and chicken extract, and suggest that while HB efficiently lures yellowjackets to the general location of a trap, it does not promote entry into traps (Figure 1). Conversely, chicken extract does not appear to lure wasps from a distance, but does promote trap entry of those present in the immediate environment (Figure 1). When placed together, traps containing only chicken caught more yellowjackets than those containing only HB (Figure 1B). However, when placed separate, neither trapped a significant number (Figure 1B). Likely, when placed together, the attractiveness of traps containing only chicken was enhanced by nearby traps containing HB. That is, HB in one trap acted as a lure for yellowjackets to the general area of the traps, while the chicken extract in the second trap acted as an entering stimulus once wasps were in the vicinity. This interaction between neighboring traps is a likely explanation for why neither trap containing a single attractant functioned efficiently when placed alone.

Surprisingly, we discovered strong negative interactions between HB and protein-derived attractants when combined. In fact, the addition of HB to traps containing chicken extract completely abrogated the ability of these extracts to promote entry of *V. pensylvanica* into traps (Figure 2). While such antagonistic interactions are rare, some are reported in the literature. For instance, the addition of acetic acid to HB blocked its ability to lure *V. vulgaris* and *V. germanica* into traps in New Zealand [22]. However, this combination previously showed synergy in attracting *V. germanica* to traps in other tests [12]. Similarly, combining highly attractive horse meat extracts with a less attractive protein source (dog food) had a sub-additive effect on *V. germanica* attraction [23]. From our assays, it appears that HB can function as both an attractant and an antagonist of chicken odors when incorporated into a trap. Perhaps HB emanating from traps attracts yellowjackets to the vicinity, but at the same time, the high concentration inside blocks the ability to sense the protein source inside, preventing wasps from efficiently entering. Others have hypothesized that a similar interaction occurs between honeydew volatiles and isobutanol in New Zealand beech forest [7]. That is, while some studies have shown isobutanol to be effective at trapping *V. vulgaris* in both Europe and New Zealand [7,24], tests conducted in a beech forest that is rich in honeydew have shown no response [22]. This discrepancy may be due to high background levels of honeydew volatiles that antagonize the attractiveness of other compounds by overwhelming or re-directing the wasp olfactory system to a more abundant food source. Laboratory electrophysiology experiments would be useful to determine whether this is truly the mechanism behind our results.

When HB is removed from the inside of the trap and placed outside, the result is enhanced trapping, with more yellowjackets visiting and entering these traps compared to those containing either or both ingredients inside (Figure 3A,B). This effect is likely dependent on an increase in HB release rate into the environment coupled with a concomitant decrease in its concentration inside the trap. Accordingly, our results show that placing a high dose of HB outside the trap attracts more yellowjackets to the area than either a high or low dose inside the trap (Figure 3C). This separated design also eliminates the antagonistic interaction between HB and chicken extract inside the trap, perhaps allowing yellowjackets to better sense the trap-entering stimulus. However, attraction to the general area of the trap appears to be dependent on HB concentration, even when it is placed inside.

While surprising, our results are not without precedent. In vespid wasps, volatile molecules can induce dramatically different responses depending on their concentration and the context in which they are sensed. For instance, the ability of tea tree oil and lemongrass oil to act as yellowjacket repellents is dose-dependent, with higher concentrations producing a stronger effect [25]. The ability of essential oils to repel also depends on their proximity to the attractant source, with repellency increasing when oils are placed closer to the attractant [26]. Some molecules are capable of eliciting opposite reactions entirely. For example, laboratory screens recently identified linalool as a repellent of *V. vulgaris* [27]. However, other studies have indicated that the addition of linalool to acetic acid enhances attractiveness to *V. maculifrons* [20]. Further, volatile spiroacetals from wasp venom are capable of attracting workers at low concentrations, but trigger an alarm response and function as repellents at concentrations greater than 1 μM [28]. Our data indicate that HB has a similar dual functionality that is dependent on both its concentration and spatial interactions with other attractants.

## 5. Conclusions

Here, we demonstrate that spatial partitioning of two common attractants used in traps against pestiferous yellowjackets significantly enhances capture of these wasps. While molecular interactions between chemical attractants are of particular interest in the field of chemical ecology, spatial interactions are often overlooked. Here, we highlight the importance of both variables in the design of baits and traps for yellowjackets. We also suggest that a greater understanding and application of the spatial and dose-dependent interactions between chemical attractants and repellents may provide benefits for the control of not only yellowjackets, but also other pestiferous insects.
